# Polymorphisms in the Tlr4 and Tlr5 Gene Are Significantly Associated with Inflammatory Bowel Disease in German Shepherd Dogs

**DOI:** 10.1371/journal.pone.0015740

**Published:** 2010-12-23

**Authors:** Aarti Kathrani, Arthur House, Brian Catchpole, Angela Murphy, Alex German, Dirk Werling, Karin Allenspach

**Affiliations:** 1 Department of Veterinary Clinical Sciences, Royal Veterinary College, University of London, Hatfield, United Kingdom; 2 Department of Pathology and Infectious Diseases, Royal Veterinary College, University of London, Hatfield, United Kingdom; 3 Department of Veterinary Basic Sciences, Royal Veterinary College, University of London, Hatfield, United Kingdom; 4 School of Veterinary Science, University of Liverpool, Liverpool, United Kingdom; University of Colorado Denver, United States of America

## Abstract

Inflammatory bowel disease (IBD) is considered to be the most common cause of vomiting and diarrhoea in dogs, and the German shepherd dog (GSD) is particularly susceptible. The exact aetiology of IBD is unknown, however associations have been identified between specific single-nucleotide polymorphisms (SNPs) in Toll-like receptors (TLRs) and human IBD. However, to date, no genetic studies have been undertaken in canine IBD. The aim of this study was to investigate whether polymorphisms in canine TLR 2, 4 and 5 genes are associated with IBD in GSDs. Mutational analysis of TLR2, TLR4 and TLR5 was performed in 10 unrelated GSDs with IBD. Four non-synonymous SNPs (T23C, G1039A, A1571T and G1807A) were identified in the TLR4 gene, and three non-synonymous SNPs (G22A, C100T and T1844C) were identified in the TLR5 gene. The non-synonymous SNPs identified in TLR4 and TLR5 were evaluated further in a case-control study using a SNaPSHOT multiplex reaction. Sequencing information from 55 unrelated GSDs with IBD were compared to a control group consisting of 61 unrelated GSDs. The G22A SNP in TLR5 was significantly associated with IBD in GSDs, whereas the remaining two SNPs were found to be significantly protective for IBD. Furthermore, the two SNPs in TLR4 (A1571T and G1807A) were in complete linkage disequilibrium, and were also significantly associated with IBD. The TLR5 risk haplotype (ACC) without the two associated TLR4 SNP alleles was significantly associated with IBD, however the presence of the two TLR4 SNP risk alleles without the TLR5 risk haplotype was not statistically associated with IBD. Our study suggests that the three TLR5 SNPs and two TLR4 SNPs; A1571T and G1807A could play a role in the pathogenesis of IBD in GSDs. Further studies are required to confirm the functional importance of these polymorphisms in the pathogenesis of this disease.

## Introduction

The healthy gut is able to regulate inflammatory responses to commensal bacteria and food antigens whilst maintaining the capacity to respond to pathogens [1], [2], [3]. This distinction relies on Toll-like receptors (TLRs) which are ideally situated on intestinal epithelia cells and recognise microbe associated molecular patterns (MAMPs) [4]. TLR2, TLR4 and TLR5 are particularly associated with recognition of lipoproteins, lipopolysaccharide and flagellin respectively [4]. A breakdown in this homeostatic response may lead to inflammatory bowel disease (IBD) in people as well as in dogs [1].

IBD is a chronic debilitating disease and occurs in both people and dogs. In dogs, it represents a group of disorders characterised by chronic gastro-intestinal signs, with histological evidence of inflammation in the lamina propria of the small intestine, large intestine or both [5]. In people, IBD consists of Crohn's disease (CD) and ulcerative colitis (UC)[6]. Inflammation associated with CD can occur in any area of the intestinal tract, although the terminal ileum is most commonly affected, whereas UC is confined to the colon [6]. CD typically involves granuloma formation and commonly involves the whole intestinal wall, while inflammation in UC is usually restricted to the superficial layers [6]. Canine IBD is a diagnosis of exclusion and in both dogs and people histopathology of intestinal sections are needed to make a definitive diagnosis [5], [6]. Immunosupressive therapy is the mainstay treatment for both human IBD and canine IBD patients [5], [6].

Although the exact aetiology of IBD is unknown it is considered a multifactorial disease with the mucosal immune system, microbiota, genetics and environment all playing a role[6], [7], [8], [9]. It is likely that human and canine IBD share similar aetiological factors as studies have already highlighted the importance of TLRs in IBD in both species. In human IBD, TLR 2,4 and 6 have been shown to be up-regulated in the intestine [10], [11], [12], whilst some studies have documented a down-regulation of TLR5[13], [14]. Similar to this, we have recently shown an upregulation of TLR 2,4 and 9 expression in the duodenum and colonic mucosa at the mRNA level in dogs with IBD of different breeds [15], [16]. Also, specific to German shepherd dogs (GSDs) with IBD, TLR4 was shown to be upregulated and TLR5 downregulated at the mRNA level [17].

In people a number of genetic polymorphisms have been reported to play a role in the pathogenesis of IBD, interestingly most of these genes are involved in the innate immune mechanism and primary defence against bacteria. Polymorphisms in the nucleotide oligomerisation domain 2 (NOD2) are considered the most important genetic susceptibility factor for CD, with approximately 20–40% of all patients carrying variants of this gene [18], [19]. However, polymorphisms in other innate immune receptors such as TLRs [20], [21], [22], the autophagy pathway (e.g. ATG16L1 and IGRM) [23], [24], cytokines (e.g. IL-10) [25] and their receptors (e.g IL23R) [26] and in human defensin (e.g. beta defensin 1) [27] genes have also been shown to be associated with IBD in people.

To date no genetic studies have been carried out in canine IBD. However, genetics are thought to play a fundamental role as certain breeds are susceptible to this disease. Retrospective analysis of the medical records at the Royal Veterinary College (RVC), UK, dating back over 10 years, revealed 555 dogs diagnosed with IBD. The GSD breed was the most common breed identified and represented 10% of the dogs diagnosed. We have previously demonstrated restriction of TLR5 allele frequencies in this breed by genetic analysis using microsatellite markers [28]. Furthermore, analysis of single nucleotide polymorphisms (SNPs) within the TLR5 gene demonstrated a significant difference in the GSD population compared to the general dog population [28]. This provides evidence that the underlying genetic make-up of this breed may be responsible for their increased susceptibility to IBD.

Comparisons of the canine and human TLR genetic sequences show a high degree of homology; this suggests that similar polymorphisms in the TLR genes seen in people with IBD may exist in dogs. Therefore, a candidate approach was used to test the hypothesis whether SNPs in genes for TLR2, TLR4 and TLR5 in GSDs are associated with IBD. We show that the TLR5 G22A, C100T, T1844C and the TLR4 SNP A1571T and G1807A SNPs are significantly associated with IBD in GSDs.

## Results

### Mutational Analysis of TLR2/TLR4 and TLR5 genes

A mutational analysis of 10 GSDs with IBD was carried out to determine the number and nature of non-synonymous single nucleotide polymorphisms (SNPs) present in the TLR2, TLR4 and TLR5 gene. This analysis revealed that there were no non-synonymous SNPs in the coding region of the TLR2 gene. Four non-synonymous SNPs were identified in the TLR4 exons; T23C (ENSCAFT00000005653), G1039A (not reported on the Ensembl web server), A1571T (ENSCAFT00000005653) and G1807A (ENSCAFT00000005653) and three non-synonymous SNPs were identified in the TLR5 exon; G22A (ENSCAFT00000018059), C100T (ENSCAFT00000018059) and T1844C (ENSCAFT00000018059). All non-synonymous SNPs identified in the TLR4 and TLR5 gene resulted in a change in the class of amino acid coded (Table 1).

**Table 1:** Amino-acid change coded by non-synonymous single nucleotide polymorphisms in the canine TLR4 and TLR5 gene.

	SNP	Amino-acid wild-type	Amino-acid change associated with SNP
**TLR4**	T23C	Valine	Alanine
	G1039A	Alanine	Threonine
	A1571T	Valine	Glutamic acid
	G1807A	Lysine	Glutamic acid
**TLR5**	G22A	Alanine	Threonine
	C100T	Arginine	Cystiene
	T1844C	Leucine	Serine

Amino-acid change coded by the non-synonymous single nucleotide polymorphisms (SNPs) identified in the TLR4 and TLR5 gene by mutational analysis of ten German shepherd dogs with inflammatory bowel disease (non-polar amino acids-valine, alanine and leucine, basic amino-acids-lysine and arginine, acidic amino-acid-glutamic acid and polar amino-acids-threonine, cystiene and serine).

### Case-control association study of TLR4 and TLR5 SNPs in German shepherd dogs

An association study was carried out to assess the significance of the non-synonymous SNPs identified by mutational analysis in GSDs with IBD. The case group consisted of 55 unrelated GSDs; 38 diagnosed with IBD at the Royal Veterinary College (RVC), UK and 17 diagnosed with IBD at the Small Animal Teaching Hospital, University of Liverpool (Table 2). The control population consisted of 61 GSDs, of which 47 dogs were diagnosed with non-inflammatory disease at the RVC, UK and 14 healthy GSDs (Table 2). The most common diagnosis for the group of GSDs diagnosed with non-inflammatory disease was neoplasia. Nineteen dogs fell into this latter category, 18 had neurological disease, three had musculoskeletal disease, two had gastrointestinal disease and there was one dog in the following aetiological categories; cardiovascular, dermatological and urogenital. Two dogs had other diseases that did not fit the above aetiological categories: one had suffered a road traffic accident, whilst the other had post-ovariohysterrectomy haemorrhage (Table 3).

**Table 2:** Signalment of cases and controls.

		Controls		Cases		P-value
		Counts	Proportions	Counts	Proportions	
**Sex**	**Male**	34	0.56	30	0.55	0.54
	**Female**	27	0.44	25	0.45	
**Age**	**1–4 years**	15	0.25	24	0.44	**0.0017**
	**5–9 years**	36	0.59	21	0.38	
	**>10 years**	10	0.16	10	0.18	

Signalment of 55 German shepherd dog (GSD) cases and 61 GSD controls included in the TLR4 and TLR5 association study of inflammatory bowel disease.

**Table 3:** Disease phenotype of controls dogs.

		Counts
**Neurological**	Degenerative myelopathy	6
	Idiopathic epilepsy	4
	Intervertebral disc disease	4
	Fibro-cartilaginous embolism	4
**Musculo-skeletal**	Hip degenerative joint disease	2
	Idiopathic generalised osteopenia	1
**Gastro-intestinal**	Pharyngeal cyst	1
	Idiopathic megaoesophagus	1
**Neoplasia**	Sarcoma	5
	Hemangiosarcoma	4
	Leukaemia	3
	Lymphoma	3
	Insulinoma	3
	Nerve sheath tumour	1
	Glial cell tumour	1
**Cardiovascular**	Idiopathic pericardial effusion	1
**Dermatological**	Peri-vascular dermatitis	1
**Uro-genital**	Protein losing nephropathy	1
**Other**	Road traffic accident	1
	Bleeding post-spay	1
**Healthy**	Blood donor	6
	Recruited for study	8

Disease phenotype of 61 German shepherd dogs (GSDs) included in the control group in the TLR4 and TLR5 association study of IBD using a SNaPSHOT multiplex reaction (case = 55 unrelated GSDs, control = 61 unrelated GSDs; 47 GSDs with non-inflammatory disease and 14 healthy GSDs).

The minor allele frequencies for all the SNPs were >10% (Table 4). The presence of Hardy-Weinberg equilibrium was tested using the exact Hardy-Weinberg test [29] using the SNPSTATS programme [30](http://bioinfo.iconcologia.net/index.php?module=Snpstats). Only three of the SNP alleles; TLR5 G22A, TLR4 T23C and TLR4 G1039A were in Hardy Weinberg equilibrium (P>0.05).

**Table 4:** Single nucleotide polymorphism allele association with inflammatory bowel disease in German shepherd dogs.

SNP	Associated allele	Minor allele freq.	P-value
**TLR5 G22A**	A	**0.112**	**5.5414e^−5^**
**TLR5 C100T**	C	**0.44**	**4.7128E^−5^**
**TLR5 T1844C**	C	**0.466**	**0.0071**
**TLR4 T23C**	T	0.274	0.1013
**TLR4 G1039A**	G	0.316	0.0926
**TLR4 A1571T**	A	**0.149**	**0.0282**
**TLR4 G1807A**	G	**0.149**	**0.0282**

Association of TLR4 and TLR5 single nucleotide polymorphism (SNP) alleles in a case-control association study of inflammatory bowel disease in German shepherd dogs (GSDs) carried out using a SNaPSHOT multiplex reaction (case = 55 unrelated GSDs, control = 61 GSDs; 47 GSDs with non-inflammatory disease and 14 healthy GSDs).

The significance of the SNP alleles was determined using the Haploview software package (version 4.1; http://www.broad.mit.edu/personal/jcbarret/haplo/). All TLR5 SNP alleles and two of the TLR4 SNP alleles (A1571T and TLR4 G1807A) were found to be significantly associated with IBD in GSDs (Table 4).

To assess the importance of the identified associated SNP alleles in their genotype forms for association in IBD, five inheritance models (co-dominant, dominant, recessive, over-dominant and additive) were applied for statistical analysis to assess which one resulted in a best fit. The best inheritance model was assessed using the Akaike information criteria (AIC) and the Bayesian information criteria (BIC), with the model with the lowest values being the best fit [31], [32].

For the two TLR4 SNPs; A1571T and G1807A the recessive model was the best fit, where two copies of T and A respectively are necessary to increase the risk of developing IBD in GSDs (Table 5).

**Table 5:** TLR4 A1571T and TLR4 G1807A genotype association with inflammatory bowel disease in German shepherd dogs.

TLR4 A1571T SNP association with IBD							
Model	Genotype	Control	Case	OR (95% CI)	P-value	AIC	BIC
Codominant	T/T	48 (80%)	38 (70.4%)	1.00	**0.0093**	154.4	162.6
	A/T	12 (20%)	10 (18.5%)	1.05 (0.41–2.70)			
	A/A	0 (0%)	6 (11.1%)	**NA (0.00-NA)**			
Dominant	T/T	48 (80%)	38 (70.4%)	1.00	0.23	160.3	165.8
	A/T-A/A	12 (20%)	16 (29.6%)	1.68 (0.71–3.98)			
Recessive	T/T-A/T	60 (100%)	48 (88.9%)	1.00	**0.0022**	152.4	157.9
	A/A	0 (0%)	6 (11.1%)	**NA (0.00-NA)**			
Overdominant	T/T-A/A	48 (80%)	44 (81.5%)	1.00	0.84	161.7	167.2
	A/T	12 (20%)	10 (18.5%)	0.91 (0.36–2.31)			
Log-additive	---	---	---	**1.99 (0.98–4.01)**	**0.047**	157.8	163.2

TLR4 G1807A SNP association with IBD							
Model	Genotype	Control	Case	OR (95% CI)	P-value	AIC	BIC
**Codominant**	A/A	48 (80%)	38 (70.4%)	1.00	**0.0093**	154.4	162.6
	A/G	12 (20%)	10 (18.5%)	1.05 (0.41–2.70)			
	G/G	0 (0%)	6 (11.1%)	**NA (0.00-NA)**			
**Dominant**	A/A	48 (80%)	38 (70.4%)	1.00	0.23	160.3	165.8
	A/G-G/G	12 (20%)	16 (29.6%)	1.68 (0.71–3.98)			
**Recessive**	A/A-A/G	60 (100%)	48 (88.9%)	1.00	**0.0022**	152.4	157.9
	G/G	0 (0%)	6 (11.1%)	**NA (0.00-NA)**			
**Overdominant**	A/A-G/G	48 (80%)	44 (81.5%)	1.00	0.84	161.7	167.2
	A/G	12 (20%)	10 (18.5%)	0.91 (0.36–2.31)			
**Log-additive**	---	---	---	**1.99 (0.98–4.01)**	**0.047**	157.8	163.2

Frequency and significance of the TLR4 A1571T and TLR4 G1807A single nucleotide polymorphism genotype in a case-control association study in German shepherd dogs (GSDs) with inflammatory bowel disease using a SNaPSHOT multiplex reaction (case = 55 unrelated GSDs, control = 61 GSDs) (OR-Odds ratio, AIC- Akaike information criteria, BIC-Bayesian information criteria).

For the TLR5 G22A SNP, the best fit model was the additive model, where having each copy of A modifies the risk of developing IBD in an additive form. As a consequence, the homozygous AA GSD has a double risk of developing IBD compared to the GA genotype (p<0.0001, OR (95% CI) = 8.51 (2.71–26.70) (Table 6). However, the AIC and BIC for the dominant model was also low and very close to the additive model and, therefore, could also be a good fit model. For both the TLR5 SNPs C100T and T1844C, the T allele was significantly protective for IBD, and exerted a dominant effect (p<0.0001, OR = 0.19 [0.09–0.44, 95% CI]: p = 7e-04, OR = 0.25 [0.11–0.58, 95% CI], respectively; Table 6). Based on univariate analysis, age and sex were not significantly associated with SNP outcome (p>0.05).

**Table 6:** TLR5 G22A, TLR5 C100T and TLR5 T1844C genotype association with inflammatory bowel disease in German shepherd dogs.

TLR5 G22A SNP association with IBD							
Model	Genotype	Control	Case	OR (95% CI)	P-value	AIC	BIC
Codominant	G/G	57(93.4%)	34(61.8%)	1.00	**1e-04**	147.9	156.2
	A/G	4 (6.6%)	20(36.4%)	**8.38(2.64–26.59)**			
	A/A	0 (0%)	1 (1.8%)	NA (0.00-NA)			
Dominant	G/G	57(93.4%)	34(61.8%)	1.00	**<0.0001**	146.3	151.8
	A/G-A/A	4 (6.6%)	21(38.2%)	**8.80(2.79–27.81)**			
Recessive	G/G-A/G	61 (100%)	54(98.2%)	1.00	0.22	163	168.5
	A/A	0 (0%)	1 (1.8%)	NA (0.00-NA)			
Overdominant	G/G-A/A	57(93.4%)	35(63.6%)	1.00	**<0.0001**	147.9	153.4
	A/G	4 (6.6%)	20(36.4%)	**8.14 (2.57–25.80)**			
Log-additive	---	---	---	**8.51 (2.71–26.70)**	**<0.0001**	145.9	151.5

TLR5 C100T SNP association with IBD							
Model	Genotype	Control	Case	OR (95% CI)	P-value	AIC	BIC
Codominant	C/C	13 (21.3%)	32 (58.2%)	1.00	**2e-04**	149.5	157.8
	C/T	27 (44.3%)	13 (23.6%)	**0.20 (0.08–0.49)**			
	T/T	21 (34.4%)	10 (18.2%)	**0.19 (0.07–0.52)**			
Dominant	C/T	13 (21.3%)	32 (58.2%)	1.00	**<0.0001**	147.5	153
	C/T-T/T	48 (78.7%)	23 (41.8%)	**0.19 (0.09–0.44)**			
Recessive	C/C-C/T	40 (65.6%)	45 (81.8%)	1.00	**0.046**	160.5	166
	T/T	21 (34.4%)	10 (18.2%)	0.42 (0.18–1.01)			
Overdominant	C/C-T/T	34 (55.7%)	42 (76.4%)	1.00	**0.019**	159	164.5
	C/T	27 (44.3%)	13 (23.6%)	**0.39 (0.17–0.87)**			
Log-additive	---	---	---	**0.41 (0.25–0.68)**	**3e-04**	151.3	156.8
Log-additive	---	---	---	**1.99 (0.98–4.01)**	**0.047**	157.8	163.2

TLR5 T1844C SNP association with IBD							
Model	Genotype	Control	Case	OR (95% CI)	P-value	AIC	BIC
Codominant	C/C	12 (19.7%)	27(49.1%)	1.00	**0.0024**	154.4	162.7
	C/T	31 (50.8%)	15 (27.3%)	**0.22(0.09–0.54)**			
	T/T	18 (29.5%)	13 (23.6%)	**0.32 (0.12–0.86)**			
Dominant	C/C	12 (19.7%)	27 (49.1%)	1.00	**7e-04**	153.1	158.6
	C/T-T/T	49 (80.3%)	28 (50.9%)	**0.25 (0.11–0.58)**			
Recessive	C/C-C/T	43 (70.5%)	42 (76.4%)	1.00	0.47	164	169.5
	T/T	18 (29.5%)	13 (23.6%)	0.74 (0.32–1.70)			
Overdominant	C/C-T/T	30 (49.2%)	40 (72.7%)	1.00	**0.0091**	157.7	163.2
	C/T	31 (50.8%)	15 (27.3%)	**0.36 (0.17–0.79)**			
Log-additive	---	---	---	**0.54 (0.33–0.89)**	**0.013**	158.4	163.9

Frequency and significance of the TLR5 G22A, TLR5 C100T and TLR5 T1844C single nucleotide polymorphism genotype in a case-control association study in German shepherd dogs (GSDs) with inflammatory bowel disease using a SNaPSHOT multiplex reaction (case = 55 unrelated GSDs, control = 61 GSDs; 47 GSDs with non-inflammatory disease and 14 healthy GSDs) (OR-Odds ratio, AIC- Akaike information criteria, BIC-Bayesian information criteria).

**Figure 1:**
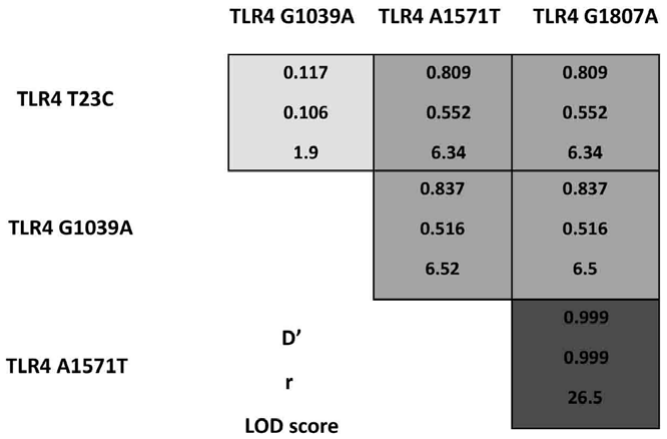
Linkage disequilibrium of single nucleotide polymorphisms in canine TLR4. Linkage disequilibrium plot of the four non-synonymous TLR4 single nucleotide polymorphisms in the German shepherd dog (GSD) population obtained from a case-control association study of inflammatory bowel disease. Sequencing information was obtained using a SNaPSHOT multiplex reaction (case = 55 unrelated GSDs, control = 61 GSDs).

**Figure 2:**
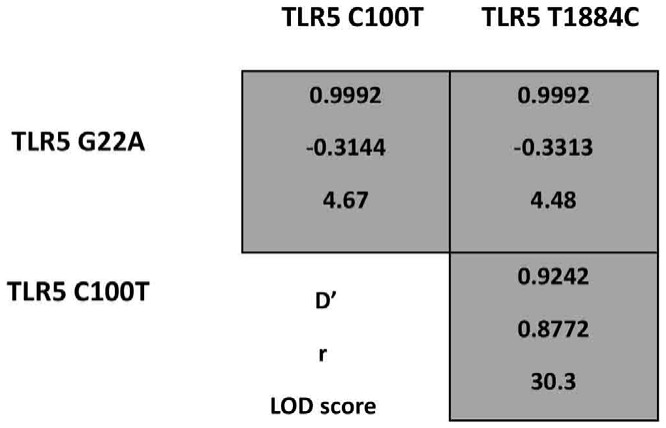
Linkage disequilibrium of single nucleotide polymorphisms in canine TLR5. Linkage disequilibrium plot of the three non-synonymous TLR5 SNPs in the German shepherd dog (GSD) population obtained from a case-control association study of inflammatory bowel disease. Sequencing information was obtained using a SNaPSHOT multiplex reaction (case = 55 unrelated GSDs, control = 61 GSDs).

The statistical correlation between different SNPs on the same gene was calculated to determine the inheritance pattern of alleles and the possible haplotype combinations. There was evidence of linkage disequilibrium (LD) between the SNPs in the TLR4 gene. TLR4 A1571T and TLR4 G1807A showed complete linkage, whereas TLR4 T23C and TLR4 G1039A showed no linkage which is not unexpected as they are present on different exons (Figure 1). There was also evidence of linkage between the three SNPs in the TLR5 gene with the TLR5 C100T and TLR5 T1844C showing the most linkage (Figure 2). There was also evidence of linkage amongst the other SNPs (Lod>3) (Figure 1 and 2).

LD analysis demonstrated significant linkage between SNPs on the same gene therefore haplotype analysis was carried out to determine their significance in IBD. The most significant haplotype combination was with the TLR5 G22A SNP, without the two protective TLR5 SNPs (C100T and T1844C) and with the two TLR4 SNPs (A1571T and G1807A) (p<0.0001). However, although the TLR5 risk haplotype (ACC) on its own without the two TLR4 SNPs was significantly associated with IBD (p = 0.029), a haplotype combination containing the two associated TLR4 SNPs without the associated TLR5 risk haplotype (ACC) was not statistically associated with IBD (p = 0.67). Of the six dogs that were homozygous recessive for the two associated TLR4 SNPs, five of these dogs had the risk haplotype for the three SNPs in their TLR5 gene (ACC).

## Discussion

The importance of TLRs in the pathogenesis of IBD in both people and dogs has been reported in the literature [10], [11], [12], [14], [15], [16]. Furthermore, SNPs in TLRs have been shown to be associated with IBD in people [20], [21], [22], [33], [34]. However, no genetic studies looking at SNPs in TLRs in canine IBD have been described. In this current study we identified three non-synonymous SNPs in the TLR5 gene (G22A, C100T and T1844C) and two non-synonymous SNPs in the TLR4 gene (A1571T and G1807A) to be significantly associated with IBD in GSDs.

Like all dog breeds the GSD has undergone selective breeding [28] and this may reflect why all the associated SNPs, except one (TLR5 G22A) were not in Hardy-Weinberg equilibrium. Additionally, for the TLR5 T1844C SNP the major allele, the T reported on the Ensembl webserver was the minor allele in the GSD population and was found to be protective for IBD. It is possible that selective breeding in the GSD population has resulted in loss of the protective effect of this major allele in the GSD population compared to the general dog population thus making them more susceptible to IBD. Similarly, for the TLR4 SNPs A1571T and G1807, the major allele reported on the ensemble webserver was the minor allele in the GSD population and was significantly associated with IBD. This may again reflect the selective breeding seen in this breed. Alternatively, it is possible that the data on the Ensembl webserver does not represent an accurate report of the major and minor alleles of the general canine population as the sequence data published is based only on a small number of breeds (Boxer and Standard Poodle).

In this study, all the SNPs associated with IBD result in a change in the class of amino acid coded. This may be significant as it could change the structural properties of the resulting TLR4 or TLR5 receptor, and therefore alter function. Interestingly, when the SNPs were protein modelled using the Simple modular architecture research tool (SMART) web server (http://smart.embl.de/)[35], [36], the location of the majority of these SNPs was found to be in significant regions. Thus, both the TLR5 T1844C SNP and TLR4 G1807A SNP were found in the leucine-rich repeat (LRR) C-terminal domain, which is the region that folds into an arc or horse-shoe shape to form the ligand binding site in TLR–receptors [37]. In addition, the TLR4 SNP A1571T was also found in a LRR region which also contributes to the ligand binding site. The TLR5 G22A SNP was found to be in the signal peptide region when mapped using the SignalP 3.0 web server (http://www.cbs.dtu.dk/services/SignalP/). The signal peptide is important in the transport of proteins and a change in the amino acid may alter the cleavage site and, therefore, may result in a functional change to the TLR5 receptor [38].

Linkage disequilibrium (LD) in dogs is up to 100 times more extensive than in human beings [39]. It is therefore, possible that the associations we found, between SNPs in TLR4 and TLR5 and the development of IBD, are not the cause of the aberrant inflammation but are in LD with the causative gene. Functional analysis of these SNPs will help to confirm if TLR4 and TLR5 play a significant role in the pathogenesis of IBD in GSDs. However, the role of TLR5 in the pathogenesis of human IBD and murine colitis has been reported in the literature. TLR5 knockout mice develop spontaneous colitis and exhibit decreased intestinal expression of TLR5 [40]. Moreover, deletion of TLR4 rescued the colitis in these TLR5 knockout mice [40]. In addition, a dominant-negative TLR5 polymorphism termed TLR5-stop protected people of Jewish ethnicity against Crohn's disease (CD) [34]. It could be speculated that patients having the TLR5 stop polymorphism may also have a mutated TLR4 thus protecting them from IBD. Moreover, In CD patients, tolerance to commensal derived flagellin is lost and serum reactivity to flagellin can be demonstrated [41]. In addition, TLR5 signalling has been shown to play a role in defensin production [42]. One study showed that TLR5 uses gangliosides as co-receptors to bind flagellin leading to increased human beta defensin-2 expression in human colon adenocarcinoma (Caco)-2 cells [43]. This demonstrates that TLR5 activation may be needed for secretion of certain defensins. The reduced gene copy number of the beta-defensin cluster seen in CD patients may therefore be due to aberrant TLR5 activation [44], [45]. Clearly, evidence has accumulated that dysregulated TLR5 function may play a role in the pathogenesis of human IBD.

Similarly, several studies have looked at the role of TLR4 in human IBD. TLR4 expression is up-regulated in both Crohn's disease (CD) and ulcerative colitis (UC) [10], [11]. In paediatric IBD patients, higher levels of TLR4 mRNA and protein were found in the inflamed colonic mucosa [12]. This confers with our findings in canine IBD [15], [16], and specifically in GSDs [17]. Epidemiological studies have also shown an association between TLR4 polymorphism and susceptibility to IBD in people. A Belgian cohort found that the allele frequency of the TLR4 A299G polymorphism was significantly higher in CD and UC patients [21]. Furthermore, the A299G SNP has been shown to be associated with reduced responsiveness following lipopolysaccharide stimulation [46]. Another TLR4 polymorphism, T399IL, was exclusively associated with UC but not CD [22].

Of the six dogs in the case population that harboured the TLR4 SNPs in a recessive genotype, five of them also had the risk haplotype for all three TLR5 SNPs (ACC). This suggests that the TLR4 SNPs may need to be present with the TLR5 SNPs in order for them to have a significant effect. It is known that TLR4 and TLR5 are able to form heterodimeric complexes and this enhances the diversity of the response to flagellin [47]. Therefore, the functional consequence of the TLR4 mutations may be acting via TLR5 signalling and so having both TLR4 and TLR5 SNPs may affect intracellular signalling after ligation of flagellin more than just the TLR5 SNPs on their own.

Our results from the mutational analysis showed that no TLR2 non-synonymous SNPs could be identified in the coding region of the gene. This finding is also mirrored in the literature as there are no published or identified non-synonymous SNPs in the canine TLR2 exon gene. Moreover, we could not detect upregulation of TLR2 mRNA in intestinal biopsies in GSDs with IBD [17]. However, the role of TLR2 in canine IBD cannot be completely eliminated as some studies, including dogs of many different breeds, have shown an up-regulation at the mRNA level in dogs with chronic enteropathies [15], [16]. This could be due to SNPs present in the promoter region of the TLR2 gene which was not investigated in this study or could be a consequence of the disease process rather than the cause.

One potential limitation of this study may be the selection and inclusion of controls having non-inflammatory disease. Although retrospective phenotyping was carried out to ensure that none of these dogs had inflammatory, infectious or immune-mediated disease, this type of inquiry can be imprecise and therefore may have led to some GSDs inappropriately being included in the control group as they may have later developed these diseases. However, to control for this, GSDs older than 4 years were recruited as controls and 75% of dogs in our control group were 5 years or older. This resulted in a significant difference in the age groups between the case and control population. However, we did have 14 completely healthy dogs in our control group, who were all over the age of 4 years and none of these dogs had the TLR5 risk haplotype (ACC) nor the two TLR4 recessive SNPs, further supporting the role of these SNPs in canine IBD in GSDs. Another potential limitation of this study was that the degree of relatedness of the dogs could not be assessed as most of these dogs were not registered with the UK Kennel Club. This may have skewed the results; however, none of the dogs belonged to the same litter. Previous studies have shown that the GSD breed is one of the most diverse dog breeds as it has retained a large range of Dog Leucocyte Antigen (DLA) haplotypes and therefore represents a breed that has been less selectively in-bred than other dog breeds [48]. Moreover, we have shown in a previous study that the same non-synonymous SNPs in the TLR5 gene are not associated with anal furunculosis in GSDs, which is another immune-mediated disease commonly seen in this breed. [28]

In conclusion, we have identified an association between three non-synonymous SNPs in the TLR5 gene (G22A, C100T and T1844C) and two non-synonymous SNPs in the TLR4 gene (A1571T and 1807A) with IBD in the GSD population. Studying the functional significance of these SNPs will not only help to confirm their importance in IBD but will also help to increase our understanding of the pathogenesis of these diseases in dogs and in man. In addition, as the pathogenesis of IBD is multifactorial, studying the nutrition and maintenance conditions of dogs with and without the mutations may help determine key triggering factors in the intestinal microbiota that are responsible for bringing about these changes and may therefore allow the development of preventative strategies not only in the dog but also in people.

## Materials and Methods

### Ethics Statement

Ethics approval for the recruitment of healthy German shepherd dogs was sought and approved from the Ethics Committee at the Royal Veterinary College (URN 2010 1035).

### Sample population

The case population included 55 GSDs; 38 were diagnosed with inflammatory bowel disease at the Royal Veterinary College, UK and 17 at the Small Animal Teaching Hospital, University of Liverpool, UK. For all cases, the diagnostic work-up was reviewed. All cases had complete medical records consisting of a detailed history at first examination and at subsequent re-examinations, haematology and biochemistry results, detailed abdominal ultrasonography report and detailed histopathology reports. All known causes of gastrointestinal inflammation including enteropathogenic bacteria were ruled out by routine haematology and biochemistry, faecal analysis, ultrasound and measurement of serum trypsin-like immunoreactivity concentration. Definitive diagnosis of IBD was based on histological evidence of an inflammatory infiltrate within the lamina propria of endoscopically obtained intestinal biopsies.

The control population consisted of 47 unrelated GSDs from the UK. These were retrospectively phenotyped, either by owner or veterinary surgeon telephone contact, to ensure they had no inflammatory, immune-mediated or infectious disease. Fourteen additional GSDs were also included in the control group; six of these were enrolled from the blood donor programme at the RVC and eight were recruited exclusively for the study. Ethics approval was granted from the Royal Veterinary College, UK for the recruitment of these eight dogs.

Blood stored in Ethylenediaminetetraacetic acid (EDTA) was collected from the DNA archive for those cases that were seen at the RVC. For the cases from Liverpool, paraffin sections of intestinal tissue were available. Residual blood from the blood donor dogs and from the eight dogs following collection of blood for folate and cobalamin concentrations as a screening test for intestinal health were collected into EDTA tubes.

### Mutational Analysis of TLR2/TLR4 and TLR5 genes

A mutational analysis of the TLR2, TLR4 and TLR5 exons was carried out, in 10 GSDs diagnosed with IBD, to determine the presence and frequency of non-synonymous SNPs in the coding regions of the genes, so that these could be investigated further in a case-control association study to determine their significance in IBD in the GSD population.

Ten cases of GSDs with IBD were selected from the 38 diagnosed at the RVC and genomic DNA was extracted from blood stored in EDTA using the Qiagen DNeasy blood and tissue kit (Qiagen, Crawley, UK) according to the manufacturer's protocol.

Full-length canine TLR2, TLR4 and TLR5 forward and reverse primers were designed using the Integrated DNA technology oligoanalyzer 3.1 programme (http://eu.idtdna.com/analyzer/Applications/OligoAnalyzer) (Table 7) based on the full sequences for canine TLR2, TLR4 and TLR5.

**Table 7:** Primers used in mutational analysis of TLR4 and TLR5.

Primer	Manufacturer		Direction	Primer sequence
Full-length TLR2	MWG Biotech (London, UK)		Forward	5′-GGA CAA TGT CAC GTG TTT TG-3′
			Reverse	5′-CGA ATC TAG GAT TTT ATT GCT GT-3′
Full-length TLR5	MWG Biotech (London, UK)		Forward	5′-TTT CCG TCC CGC AGG ATC ATG-3′
			Reverse	5′-ACC CGT GCA AAT GGC AAG AG-3′
Full-length TLR4	MWG Biotech (London, UK)	TLR4 Exon 1	Forward	5′-TTC CTC TTG CCC CTT AA CTC-3′
			Reverse	5′-GCC ATG TAA CCA TGA ACT GT-3′
		TLR4 Exon 2	Forward	5′-GAT GGA TGG ATG GAC AGA CC-3′
			Reverse	5′-TTC ACA GAT GAG GCA ATG GG-3′
		TLR4 Exon 3	Forward	5′-ATA CTG AAT CTG TGG GGC TT-3′
			Reverse	5′-ATT TGG TGG AAG CAT CCA CCT-3′

Primers used in the TLR2/TLR4 and TLR5 mutational analysis.

Easy A 2X master mix (Stratagene, California, USA) was used for polymerase chain reaction (PCR) with reactions heated to 95°C for 10 min, followed by 30 cycles of 95°C for 1 min, 55°C for 1 min and 72°C for 3 mins then a further 72°C for 7 mins.

Full-length PCR products were electrophoresed with 6X orange loading dye (Fermentas, UK) on a 1% agarose/safeview nucleic stain (NBS Biologicals, Huntington, UK) gel in 1X Tris-Borate EDTA (TBE) buffer using the power pack FB 300 (Fischer Scientific, UK). The gels were visualised under UV light (Ultraviolet Transilluminator, UVP, California,USA), and the bands cut out and placed into separate eppendorf tubes. DNA was extracted using the Qiagen mini-elute extraction kit (Qiagen, Crawley, UK), according to manufacturer's instructions.

Gel purified PCR products were sent to Geneservice, Cambridge, UK for sequencing using sequencing primers specifically designed to cover the entire sequence of the exons (Table 8).

**Table 8:** Sequencing primers used in mutational analysis of TLR4 and TLR5.

	Manufacturer		Primer	Sequence
TLR2	MWG Biotech (London, UK)		TLR2 forward	5′-GGA CAA TGT CAC GTG TTT TG-3′
			TLR2 internal forward	5′-CAT TTG GAC ACT TTC CAC-3′
			TLR2 internal reverse	5′-AAG ACT TTG GCC AGT GCT-3′
			TLR2 reverse	5′-CGA ATC TAG GAT TTT ATT GCT GT-3′
TLR5	MWG Biotech (London, UK)		TLR5 forward	5′-TTT CCG TCC CGC AGG ATC ATG-3′
			TLR5 internal forward	5′-AGA CCC TGG ACG TGT CTA A-3′
			TLR5 internal reverse	5′-CCA TGG AGA CAG AGG ACA-3′
			TLR5 reverse	5′-ACC CGT GCA AAT GGC AAG AG-3′
TLR4	MWG Biotech (London, UK)	Exon 1	TLR4 exon 1 forward	5′-TTC CTC TTG CCC CTT AA CTC-3′
			TLR4 exon 1 reverse	5′-GCC ATG TAA CCA TGA ACT GT-3′
		Exon 2	TLR4 exon 2 forward	5′-GAT GGA TGG ATG GAC AGA CC-3′
			TLR4 exon 2 reverse	5′-TTC ACA GAT GAG GCA ATG GG-3′
		Exon 3	TLR4 exon 3 forward	5′-ATA CTG AAT CTG TGG GGC TT-3′
			TLR4 exon 3 internal forward	5′-GGT CTG GCT GGC TTA AAG-3′
			TLR4 exon 3 internal reverse	5′-CAA CTT CCA CCA AGA GCT G-3′
			TLR4 exon 3 reverse	5′-ATT TGG TGG AAG CAT CCA CCT-3′

Sequencing primers used in TLR2/TLR4 and TRL5 mutational analysis.

Sequence data was compared to the canine genome (www.ensembl.org/Canis_familiaris) and CLC DNA Workbench version 5.1 (CLC Bio) used to identify non-synonymous SNPs present in the genes.

### Determining the allele frequency of TLR4 and TLR5 SNPs in healthy GSDs and those affected with IBD using SNaPSHOT multiplex reaction

Non-synonymous SNPs identified by mutational analysis in the TLR2, TLR4 and TLR5 exon genes were analysed further in a case population consisting of 55 GSDs with IBD and a control population consisting of 61 GSDs; 47 with non-inflammatory disease and 14 healthy. Genotyping of all SNPs was carried out by primer extension using the ABI PRISM SNaPSHOT Multiplex Kit (Applied Biosystems).

Genomic DNA was extracted from blood stored in EDTA from the remainder of the 28 IBD cases seen at the RVC and from the 61 control GSDs using the Qiagen DNeasy blood and tissue kit (Qiagen, Crawley, UK) according to the manufacturer's protocol. This kit along with the manufacturer's recommendations was also used to extract genomic DNA from the paraffin sections from the 17 cases diagnosed with IBD at the University of Liverpool.

PCR was carried out on all samples using amplification primers to yield a fragment size containing the SNPs that was less than 1200 base pairs (Table 9). Immolase DNA polymerase (Bioline, London, UK) was used for PCR with reactions heated to 95°C for 10 min, followed by 35 cycles if 95°C for 1 min, 52°C for 1 min and 72°C for 2 mins, then a further 72°C for 7 mins.

**Table 9:** Fragment amplification primers used in SNaPSHOT reaction of TLR4 and TLR5.

Amplification primer	Manufacturer	Forward sequence	Reverse sequence
TLR4 Frag 1 (containing SNP T23C)	MWG Biotech (London, UK)	5′-TCC CTC TTG CCC CTT AA CTC-3′	5′-GCC ATG TAA CCA TGA ACT GT-3′
TLR4 Frag 2 (containing SNP G1039A, A1571T and G1807A)	MWG Biotech (London, UK)	5′-TCA AGG TCT GGC TGG CTT-3′	5′-CCA CAG GAG CTT TTC ATC-3′
TLR5 Frag 1 (containing SNP G22A and C100T)	MWG Biotech (London, UK)	5′-GAT TGA GTG ACG GCA AAC-3′	5′-CCT GTT GAT CTT GTT GTG-3′
TLR5 Frag 2 (containing SNP T1844C)	MWG Biotech (London, UK)	5′-ACA ACA AGT TCA TCT GCG-3′	5′-CTC CTC GAA GCA CAG GTT-3′

TLR4 and TLR5 fragment amplification primers used in SNaPSHOT reaction.

Five micro-litres of PCR product from each sample was added to 2 µL of ExoSAP-IT (United States Biochemical (USB), USA) and mixed thoroughly and incubated for an hour at 37°C and then at 75°C for 15 minutes.

Sequencing primers of different lengths for the SNaPSHOT reaction were designed to stop just one base before the SNP (Table 10). All SNaPSHOT primers were diluted to give a concentration of 0.2 µL as a working solution. For multiplexing 0.2 µM concentration of each primer was used.

**Table 10:** Sequencing primers used in SNaPSHOT reaction of TLR4 and TLR5.

Primer	Manufacturer	Forward sequence
TLR4 SNaPSHOT SNP T23C	MWG Biotech (London, UK)	5′-ATG TCT CCT ACC CGC CTG G-3′
TLR4 SNaPSHOT SNP G1039A	MWG Biotech (London, UK)	5′-GCG GCG AAC TTA GAA CAA TTT CCC-3′
TLR4 SNaPSHOT SNP A1571T	MWG Biotech (London, UK)	5′-GAC GAC GAC GAC CAC TTC CTA AAC TTC AGG-3′
TLR4 SNaPSHOT SNP G1807A	MWG Biotech (London, UK)	5′-GGA ATG TAT GCA TGC ATC ACA CCA TTT GTT CAA CTT-3′
TLR5 SNaPSHOT SNP G22A	MWG Biotech (London, UK)	5′-GAC TGA CTG ACT GAC TGA CTG ACT CCG CCA GCT GGG CCG C -3′
TLR5 SNaPSHOT SNP C100T	MWG Biotech (London, UK)	5′-ACT GAC TGA CTG ACT CTG GCT GAG GTT GCA GGA GC-3′
TLR5 SNaPSHOT SNP T1844C	MWG Biotech (London, UK)	5′-GAC TGA CTG ACT CTG CGC CTA CCC CAG CT-3′

TLR4 and TLR5 sequencing primers used in SNaPSHOT reaction.

The SNaPSHOT multiplex reaction mix (ABI prisms, Applied Biosystems) was thawed on ice. For each reaction the following was added to a 96-well plate: 4 µL undiluted DNA ExoSAP-IT cleaned template, 1 µL SNaPSHOT SNP primer mix, 2 µL SNaPSHOT reagent mix, 2 µL sequencing buffer (ABI prisms, Applied Biosystems) and 1 µL MWB.

The plate was incubated at 96°C for 10 seconds, 50°C for 5 seconds and at 60°C for 30 seconds. This was repeated for 25 cycles.

Once the sequencing reaction was complete the samples were removed from the thermal cycler and 1 µL of shrimp alkaline phosphatase (SAP) (USB, USA) was added to each well. The plate was then incubated at 37°C for one hour and the enzyme deactivated by incubating at 75°C for 15 minutes.

A master mix was made up per reaction well of 9.0 µL Hi-Di Formamide (ABI prisms, Applied Biosystems) and 0.5 µL Gene-Scan 120 Liz size standard (ABI prisms, Applied Biosystems). Half a micro-litre of SAP cleaned SNaPSHOT product was aliquotted into a red fisher plate (Fisher Scientific, UK). Nine and a half micro-litres of master mix was added to each well. The plate was covered with a 96 well grey septa and spun briefly in a centrifuge. The plate was then denatured at 95°C for 3 minutes and snap cooled on ice for 5 minutes and placed in ABI Prism 3100 genetic analyzer for electrophoresis.

Electrophoresis results were analysed using Genemapper (version 3.2.1) software (Applied Biosytems). Allele frequency, association and linkage disequilibrium data for all the SNPs were statistically assessed using Haploview software package (version 4.1; http://www.broad.mit.edu/personal/jcbarret/haplo/). The calculation of Hardy-Weinberg equilibrium and further genotype and haplotype associations for all the SNPs were carried out using SNPSTATS programme (http://bioinfo.iconcologia.net/index.php?module=Snpstats). Statistical significance was set at P<0·05.
